# Metals and Disease: A Global Primary Health Care Perspective

**DOI:** 10.1155/2011/319136

**Published:** 2011-10-05

**Authors:** Ravinder Mamtani, Penny Stern, Ismail Dawood, Sohaila Cheema

**Affiliations:** ^1^Global and Public Health, Weill Cornell Medical College in Qatar, P.O. Box 24144, Doha, Qatar; ^2^Preventive Medicine, Department of Population Health, North Shore University Hospital, 175 Community Drive, 2nd Fl. Great Neck, NY 11021, USA; ^3^Occupational Health and Safety Unit, Ethekwini Municipality, P.O. Box 5892, Durban 4000, South Africa

## Abstract

Metals are an important and essential part of our daily lives. Their ubiquitous presence and use has not been without significant consequences. Both industrial and nonindustrial exposures to metals are characterized by a variety of acute and chronic ailments. Underreporting of illnesses related to occupational and environmental exposures to chemicals including metals is of concern and presents a serious challenge. Many primary care workers rarely consider occupational and environmental exposures to chemicals in their clinical evaluation. Their knowledge and training in the evaluation of health problems related to such exposures is inadequate. This paper presents documented research findings from various studies that have examined the relationship between metal exposures and their adverse health effects both in developing and developed countries. Further, it provides some guidance on essential elements of a basic occupational and environmental evaluation to health care workers in primary care situations.

## 1. Introduction

Metals are ubiquitous. They are an important and essential part of our daily lives. Their benefits are many. They have contributed immensely to rapid advances in health care, information technology, telecommunications, construction, and other sectors of industry. Additionally, metals such as iron, copper, zinc, and molybdenum are essential for innumerable biological processes and enzymatic reactions that occur in the human body. But their presence in our environment presents health risks and hazards. They have the potential to cause acute toxicity. Additionally, through their insidious mode of action metals are notorious for promoting many chronic conditions including carcinogenesis. They present serious challenges. The public health professionals, safety staff, and policy makers who develop policies and implement safety programs to protect the health of workers and other members of society must become conversant with the hazards that metals present and develop programs aimed at preventing and controlling human exposure. Additionally, it is crucial that primary and other basic and frontline health workers possess adequate knowledge and skills to evaluate and manage the exposures and counsel patients. 

Here is some basic information about metals. All chemical matter consists of pure chemical substances called elements. There are three types of elements: metals, nonmetals, and metalloids. Metals, which account for about two-thirds of all elements, are good conductors of both electricity and heat. Examples of metals include iron, copper, mercury, and zinc. Unlike metals, nonmetals are poor conductors of electricity and heat. Examples of nonmetals are hydrogen, carbon, and halogens. Metalloids, also called semimetals, have intermediate properties. They are semiconductors and are vital in computers and industry, generally. Arsenic, antimony, and bismuth are classical examples of nonmetals. Alloy is a mixture of two or more elements of which metal is a primary element. Steel, of which iron is a primary constituent, is an alloy. Trace metals are those that make up less than one gram of the human body. Copper and zinc are examples of trace metals. All metals can exert toxic effects. Dose, route of exposure, and duration of exposure determine whether a metal can exert its toxic effect or not. 

Metals are also classified according to their atomic weights: heavy and light metals. Heavy metals, such as cadmium and mercury, have higher atomic weights. Many of them are toxic. But there are other heavy metals such as molybdenum, which are essential for normal human physiology. So, it is not true that all heavy metals are toxic. Light metals can also be toxic. Beryllium is a case in point. 

In general, exposures to metals occur in two ways: one, via their presence in the environment (air, food, water, and soil) and two, by undergoing transformation in their structure. In such transformations metals can exhibit a higher level of toxicity. Examples of transformations include mercury changing to more toxic methyl-mercury and the increasing concentration of metals moving up the food chain on account of their binding capacity to sulfhydral groups present in proteins [1]. The exposure to metals can occur at work, home, or in the community environment. Occupational exposure to metals is of serious concern and is discussed later in the paper. 

Why should we be so concerned about metals and metalloids? There are several reasons for the concern: recent increase in production of chemicals (including metals), serious health risk associated with metal exposures, and underreporting of medical problems related to occupational and environmental exposures to chemicals, to name a few [2–4]. 

In this paper, we discuss the growing worldwide concern surrounding exposure to chemicals and metals. We report on exposure incidents and findings of some studies that have examined the relationship between metal exposures and their adverse health affects both in developing and developed countries. Special situations such as exposures among children and in home environments are also discussed. Relevant toxicological aspects of hazardous metals such as arsenic, lead, and mercury and their effects on human health are summarized. Finally, we provide some guidance on essential elements of a basic occupational and environmental evaluation in primary care situations. Our review is aimed at basic and primary health care workers who provide care to individuals exposed to chemicals but are unfamiliar with basic clinical aspects of occupational and environmental health evaluations and toxicology [5]. Due to the diverse nature of the subject, we are unable to discuss every clinical aspect of toxicology of metals and their impact on health. However, our discussion sensitizes health care workers to essential basic information and initial steps they can take to integrate chemical exposure assessments in their day-to-day clinical work.

## 2. Growing Concern over Exposure to Chemicals and Metals

The production of chemicals (including metals and their variants) around the world has increased dramatically in recent years. It has been reported that there has been a 10-fold increase in the global output of chemicals worldwide [2]. This trend is likely to escalate in years ahead. Many experts believe that with recent advances in metal technology and increased contamination of the environment due to energy production, the potential for exposure to metal toxicity has increased in recent years. What is even more worrisome is the production of chemicals in many developing countries where public health laws are either weak or insufficient to protect the health of their workers and residents. 

Chemicals including metals account for significant mortality and morbidity. The World Health Organization (WHO) estimates that more than “25 percent of total burden of disease is linked to environmental factors including exposure to toxic chemicals.” It is believed that lead, a heavy metal, for example, is thought to be responsible for 3 percent of cerebrovascular disease burden worldwide [2]. In a recent carefully conducted analysis, Pruss-Ustun and colleagues of WHO have estimated that 4.9 million deaths (8.3 percent of total mortality worldwide) are attributable to environmental exposure and inappropriate management of selected chemicals [3]. In communities of low-income nations, particularly those with marginal resources, the consequences of such exposures can be grave. A case in point is the serious risk that arsenic-contaminated groundwater presents to many people living in developing countries. In Bangladesh, for example, where half the population is exposed to arsenic-contaminated drinking water from tube wells, the risk of adverse effects is significantly high. It has been reported that in 2001 arsenic-contaminated water caused 9,100 deaths and 125,000 disability adjusted life years (DALYs) in this Asian nation [4]. Disability adjusted life years is a measurement that “combines the burden due to death and disability in one single index. One DALY can be thought of as 1 lost year of healthy life” [2].

One concern that merits attention is the failure by health care workers to recognize many occupational and environmental health-related problems [6]. Reasons for this, among other factors, include lack of training of health care providers and medical students in occupational and environmental medicine, low level of suspicion for work- and environment-related health problems, and inadequate management or failure of management of such problems [5, 7–9]. In general, health care workers who take care of their patients have limited knowledge and skills in evaluating those patients who suffer from occupational health-related problems [7].

## 3. Review of the Literature

In both the developing and developed countries the ubiquitous presence and use of heavy metals have not been without significant consequences. The most highly developed nations of the world have been both the primary beneficiaries of the advances spurred by industrialization and the sometimes-unwitting source of problems that have accompanied these advances. Many nations of the developing world, such as China and India, are rapidly becoming epicenters for all types of manufacturing and industrial activities and are expected to suffer the inevitable human and environmental consequences of unbridled industrial expansion. 

In recent years, interest in heavy metal exposures has further intensified among populations previously thought to be less vulnerable to such exposures by virtue of living in the more developed communities. Many of these nations are often in the forefront of research and the development of regulatory protections to benefit both individuals with potential occupational exposures and those who may be environmentally exposed. Additionally, because of increasing international trade, unexpected points of exposure intersection have been identified, resulting in enormous concern. The concerns over unacceptable levels of metal concentration in certain products sold to consumers are legitimate. Increased governmental regulations and disclosure of product contents on the part of manufacturers are required.

### 3.1. More Developed Countries

Metals are of particular concern as they play essential roles in the manufacture of thousands of products destined for use in the developed world and beyond. The negative aspects of occupational and environmental exposures to heavy metals continue to be researched and documented. Both industrial and nonindustrial exposures are characterized by a variety of acute and chronic ailments, the specifics of which depend on the metal in question as well as how it is being handled [3, 10]. 

Exposure to mercury (in various elemental, inorganic, and organic forms) has long been linked to neurological problems, to acute toxicity associated with inhalation of mercury vapor, to cardiovascular, renal, and reproductive problems [11] and has been implicated as a possible contributing factor to chronic immune disorders. Although its risks have been well characterized and, as a consequence, has been phased out of some once-common usages particularly in developed countries, it continues to be utilized in industries as diverse as cosmetics, lighting, electrochemistry, and pharmaceuticals. In addition to industrial concerns, an ongoing mercury-related issue centers on methyl mercury's presence in the food chain, specifically in fish and other seafood. 

Stringent measures to control industrial contamination of waterways are only part of the solution to mercury contamination. According to the US Department of Energy, a certain amount of the mercury found in the atmosphere results from the combustion of coal and other fossil fuels [12]. Through precipitation, atmospheric mercury eventually finds its way into surface water where bacterial action transforms inorganic mercury into the toxic methylmercury, which accumulates in the food chain. Recent genomic research has further illuminated potential pathways for the bacterial mercury methylation mechanisms [13].

Over the recent past, considerable interest was stirred in the health problems attributed to mercury-containing dental amalgams. Researchers have been unable to confirm the presence of amalgam-related disease in persons exposed occupationally or otherwise to mercury-containing amalgams [14, 15]. Thus, the practice of using amalgams continues although the use of resin composites has grown as techniques to strengthen these ceramic-plastic composites have improved. 

Though known for thousands of years thanks to its many useful applications, lead's toxic properties have become more widely recognized over the past 150 years (though it is believed that even the Romans were aware of some of its less salubrious qualities). Writing in his monumental work, *De Architectura*, Vitruvius, who lived more than 2000 years ago, strongly advised against the use of lead for water pipes and noted that those who worked with lead looked unhealthy [16]. Nevertheless, lead has been used through the ages for a myriad of purposes including piping, soldering, ceramic glazes, paints, glassware, construction, bullets, batteries, and more. 

According to the Agency for Toxic Substances and Disease Registry (ATSDR), in the US, significant numbers of workers are regularly exposed to lead as a direct consequence of their employment [17] in industries such as smelting and lead battery production. In addition, ATSDR notes that nonoccupational settings also may provide sources of exposure from old leaded paint surfaces, to water from lead-contaminated pipes and to cigarettes. Despite the fact that leaded paints have been banned in various countries around the world beginning in 1909 (when Austria, Belgium, and France prohibited the use of white lead interior paint) and that leaded gasoline began to be phased out in the early 1970s [18], lead remains a potent force in the environment.

In the developed world, grave concerns continue over the effect of lead exposure on children's cognitive abilities. Since 1971, blood lead levels requiring intervention for children have been reduced from 40 *μ*g/dL to the current <10 *μ*g/dL. But ongoing research suggests that there is no “safe” blood level in children and that cognitive impairment may occur at much lower levels [19, 20].

Cadmium, a human carcinogen, became a more visible presence on the heavy metal stage as a component in nickel-cadmium batteries as well as its use in a number of industrial applications including automotive and aircraft industries as well as its presence in plastics and paints. Following the European Union's restriction of cadmium use in batteries in 2008, cadmium dealers began to seek out other markets for the material as demand was reduced [21]. 

Beryllium is used for aerospace components, precision instruments and also brings with it a variety of health risks. Dermatologic, respiratory, and malignant diseases may all result from unprotected exposure to beryllium [10]. Its use in electronic components has made it a key heavy metal problem in terms of waste management, as it joins other heavy metals in less environmentally sensitive landfills and incinerators.

Between 2007 and 2011 hundreds of news reports and scholarly papers were published detailing concerns over toxic materials contained in various products including children's toys and jewelry. In 2011, US researchers determined that inexpensive jewelry (often meant for children and originating in manufacturing facilities outside the United States) contained cadmium concentrations some one hundred times the exposure limit [22]. Testing commissioned by the Associated Press in 2010 found that objects such as illustrated drinking glasses featuring popular characters contained excessive lead concentrations [23]. These and other revelations regarding exposure in children to potentially damaging materials continue to be of major concern.

One other issue that merits some discussion is related to the use of metals in the developed world with profound implications for the developing world. The disposal of electronic waste is a case in point. Contained within the materials dubbed “e-waste” are metals including mercury, lead, beryllium, and cadmium. These metals are contained within components of many electronic devices currently in extraordinarily wide distribution throughout the world today. In the developed world, many of these obsolete devices find their way into landfills and incinerator facilities. A significant amount of e-waste, however, is transported to the developing world [24, 25] as some waste management companies try to circumvent increasingly stringent policies designed to protect the environment by requiring recycling and/or predisposal extraction of toxic materials. The United Nations Environment Programme (UNEP) cautioned in its 2010 report “Recycling—from E-Waste to Resources” that China, for example, improperly handles much of this waste, utilizing unregulated incinerating techniques to recover valuable metals. Moreover, though China has banned e-waste imports, these materials continue to arrive and are handled together with China's own dramatically high levels of e-waste—over 2 million tons annually (only the US produces more e-waste.) [26, 27]. A number of other countries in the developing world are also at risk for damaging effects as a consequence of e-waste, and the United Nations has made this problem an important and ongoing focus of UNEP and other relevant agencies.

### 3.2. Less Developed Nations

Developing countries are undergoing rapid industrial development. Occupational and environmental exposures in many communities of these nations are common and have been extensively reported in medical literature. Most experts agree that government regulations and laws are weak in many developing countries and do not always provide adequate protection to the public and workers. The use of obsolete and outdated technology further contributes to these exposures. A blind eye is turned to the regulations and requirements for protecting human health and the environment in order to gain rapid economic growth. 

Despite frequent reports of their adverse health effects, the chemical exposures continue to present challenges in the developing world. Heavy metal exposures of lead, arsenic, and mercury remain the main threats to human health [26]. Dental amalgam, contaminated fish and food, and fertilizers are possible sources of mercury exposure in many communities [27]. In 1961 in Pakistan, Agrosan GN (a mixture of phenyl mercury acetate and ethyl mercury chloride) poisoning was reported due to eating of the seed wheat, which had been treated with the chemical. The incident resulted in several fatal cases [28]. In rural Iraq, in early 1972, an epidemic of methyl mercury poisoning occurred after ingestion of homemade bread prepared from wheat. The bread had been treated with a methyl mercury fungicide [29]. Mercury is also used in large amounts in the gold mining industry thereby presenting health risk to the industry workers. It has been reported that the dramatic rise in the price of gold has led to increased illegal mining, often in developing countries [30]. This process is unregulated and raises serious concerns. 

Arsenic, another metal toxicant, is commonly used in the manufacture of wood preservatives and pesticides, widely used in the developing world. In the general population, exposure to arsenic is predominantly through food intake and drinking water. The exposure through food is more common; however, in some countries presence of inorganic arsenic in drinking water has been reported to be a major source of exposure. In several countries around the world like Chile, Bangladesh, and China inorganic arsenic is present in groundwater that is primarily used for drinking [31]. Also, with arsenic, there is a recognized interaction with smoking that increases the risk of cancer. 

Developing regions bear the brunt of the highest burden of lead exposure. Lead is commonly found in paint, lead-tainted soil, and battery manufacturing industry. The general population is exposed to lead pollution roughly in equal proportion from food and air. A major source for cadmium exposure is cigarette smoking, which is widespread in the developing world. However in the nonsmoking general population, food remains the most important source of cadmium exposure in most countries.

Despite its rapid industrialization, Thailand continues to grapple with the problem of lead pollution. In one pilot study, garage workers were found to have significantly high levels of lead in their blood [32]. Lead exposure monitoring amongst the high risk workers in Thailand such as in mechanics and dye sprayers has been clearly overlooked, and specific control measures for these high risk occupations have not been set [32]. In another study from Taiwan investigators observed that the occupational lead exposure, herbal drug use, and drinking water from certain sources are important risk factors for high blood lead in the general population [33]. 

In an extensive review of human exposure to lead in Chile, the investigators observed that lead pollution in Chile persists [34]. They identified city and home soil, as well as soil near the highways as major sources of this pollution. Clusters of exposure among certain occupational groups were also noted. 

In many countries, leaded gasoline continues to pose as a major health exposure problem with autos and trucks emanating leaded exhaust fumes and other contaminants into the environment [35, 36].

Even though overall awareness in regards to metal toxicity has increased worldwide, most experts agree that chemical exposure incidents in many communities either go unnoticed or when noticed the attention they receive is marginal. Developing countries need to develop stringent policies and enforce public health laws to protect the public from chemical exposures. Additionally, more public health research is warranted to assess the magnitude of the problem and identify all the sources of exposures.

## 4. Special Considerations

### 4.1. Children

Children are more susceptible to environmental hazards than adults due to several reasons. Generally, children drink more water, breathe more air, and eat more food per unit weight as compared to adults. Children spend a significant amount of time on the ground and floor. All of these reasons increase a child's opportunity for exposure to metals. Additionally, since normal childhood development includes hand-to-mouth behavior, children are more likely to come into contact with metal toxicants in dust, carpets, or in the soil. Due to differences in children's metabolism and behavior, and poorly developed mechanisms for detoxification, children may not be able to get rid of the toxic substances efficiently. This may result in higher levels of exposure among children within the same environment when compared with adults [37]. Since children play more outdoors they become more vulnerable to short-term illness and other types of derangements from ambient air pollution [38].

Fresh water and ocean fish may contain large amounts of mercury. Excessive consumption of fish by pregnant women and children lead to significant mercury exposure. “The developing fetus and young children are thought to be disproportionately affected by mercury exposure, because many aspects of development, particularly brain maturation, can be disturbed by the presence of mercury” [39]. This was exemplified in Minamata Bay, Japan in the 1950s where large quantities of mercury was discharged into the bay, and subsequent ingestion of the contaminated fish by mothers during pregnancy led to 41 deaths and at least 30 cases of profound brain injury in infants [40]. Clearly minimizing mercury exposure is essential to optimal child health. 

In many countries, children continue to be chronically exposed to a range of common pollutants like lead and organic pollutants at background levels [41]. Children absorb a larger percentage of inhaled and ingested lead than adults do. Furthermore, inorganic lead can penetrate the blood-brain barrier in children but not in adults. This is so because the barrier is not fully developed in children [42]. Due to these reasons, children are highly susceptible to lead exposure and subsequent nervous system damage [42]. It is estimated that the prevalence of elevated levels of blood lead in children worldwide is approximately 40%, with children in developing countries at greater risk of exposure than those in the developed countries [43]. 

Cadmium, another metal of concern, has a long half-life of 10–30 years in bones and kidneys. As a result children exposed to the metal end up suffering more from cadmium exposure than adults. Many plants, especially rice plant, can absorb cadmium from soil. Also cadmium is an ingredient of tobacco and tobacco smoke. In many Asian developing countries or communities where rice is a staple food and prevalence of smoking is widespread, cadmium exposure presents a serious threat to the health of children [44].

It should be mentioned that soil pica, which is ingestion of high amounts of soil, presents a serious risk of metal toxicity to children who engage in this activity. Soil in many communities can be contaminated with lead paint, chips, pesticides, and take—home contaminants such as mercury. 

Even though all children are affected by environmental exposures, there is a disproportionate risk to children living in poverty or in certain ethnic and minority communities. Poverty compounds the risk of exposure and impending health effects since it is clearly associated with inadequate housing (with flaking lead-based paint leading to lead exposure), poor nutrition, and inadequate access to healthcare [44].

### 4.2. Reproductive Hazards

Exposure to chemicals and metals can impair reproductive processes in men and women. The data from the US [45] suggests that the prevalence of reproductive problems related to environmental toxicants is rather low. Data from low-income nations on the subject is inadequate to make any definitive comments. 

While employed women are more likely to have better pregnancy outcomes than those who are not, there are certain occupations and exposures that cause adverse pregnancy outcomes. Exposure to toxins in the first trimester can be serious. Pregnant women at risk can develop lead exposure of the fetus which can also cause neurobehavioral and low birth weight problems. Adverse reproductive effects such as spontaneous abortions and birth defects due to lead and mercury exposures have been well documented [45]. Adverse reproductive effects in men have also been reported. Metals such as lead and mercury are known to cause spermatotoxicity.

### 4.3. Domestic Exposures

There are several sources of chemical exposures in domestic environment. Hobbies such as painting and welding, cleaning agents, second hand smoke, water supply, and job situations of household members (take home contamination) are examples of such sources. Take home contamination, which is transmission of chemicals from workplace to homes, is often overlooked. This type of exposure affects the immediate family members of the contaminated worker. The clothes, shoes, and other personal belongings of these workers should never be brought home for washing and/or cleaning. Small children are very susceptible to such exposures. There have been several reports of lead contamination among children of workers who are exposed to lead at their workplace [46]. 

One other issue that deserves special mention is the exposure to metals through the use of oral and topical herbal remedies commonly used worldwide. The two alternative systems of medicine, which rely on extensive use of herbs, are Ayurveda, a traditional healing system practiced in India, and the Traditional Chinese Medicine (TCM), commonly used in China. Many patients in the Western nations also use both systems. 

It has been documented that some Ayurvedic herbal preparations, which contain metals, have been associated with adverse health effects. In one Center for Disease Control and Prevention report 12 cases of lead poisoning were found to be associated with the use of Ayurvedic remedies [47]. In two other studies Saper et al. have shown that some of these herbal preparations, which are available in the US, may contain potentially harmful levels of arsenic, mercury, and lead [48, 49]. TCM herbal remedies are also known to be associated with metal contamination and toxicity. Metals such as lead and arsenic have been implicated in these negative outcomes [50, 51].

## 5. Metals of Concern

It is evident that many metals present serious risk to human health. In this section we provide a brief summary of lead, arsenic, and mercury, which have been implicated in various occupational and environmental exposures around the world resulting in serious morbidity and mortality. They are the main threats to human health. Examples of other metals that can produce adverse health effects, but not mentioned in this discussion, include cadmium, cobalt, zinc, and aluminum. 

Arsenic is a metalloid, which cannot be destroyed. It is present in the Earth's crust. Arsenic compounds are classified as either organic or inorganic. Arsenic compounds have no smell or taste, and therefore it is hard to detect their presence in food, water, or air. Inorganic arsenic which is found in soil and rocks is mainly used as a wood preservative to prevent its rotting. When attached to small particles it can stay in air for several days and carried to distance sites. It is also present in very small amounts in potable water, wines, and seafood [52–54]. Arsenic can be ingested or inhaled; its main route of excretion is via urine. 

Lead is a grayish blue metal, which exists in the Earth's crust. It can leach into drinking water and enter food items. Its corrosion resistance and low melting point properties make lead an attractive substance for its extensive use in pipes and batteries. Its primary route of exposure is ingestion by way of drinking water, lead-containing paint or chips and contaminated dust. It is excreted in urine and feces. Children are particularly susceptible to its toxicity [54, 55]. 

Mercury exists in three forms: elemental (metallic), organic (methyl mercury), and inorganic. Methyl mercury is the most toxic form and exerts its effect by accumulating in the central nervous system. It is formed by microorganisms in soil and water. It is found in fish; swordfish and sharks have the highest level of mercury in their bodies. Elemental mercury, also called quicksilver, is found in household items such as thermometers, fluorescent light tubes/bulbs, and thermostats. It is slowly absorbed and less toxic than methyl-mercury [56, 57]. 

Adverse health effects due to metal exposures have been extensively documented [3, 10, 51–57]. Types of exposure to arsenic, lead and mercury, and their health effects appear in Table 1.

## 6. Incorporating Occupational and Environmental Assessment in Primary Care: A Global Viewpoint

It is evident that metals present serious and significant health risks. The hazards that metals present are a function of the toxic properties of metals: duration, dose and route of exposure, and health history of the individuals exposed to them. Controlling and preventing metal exposures will require a multidisciplinary and integrated strategy warranting close collaboration between the government, employer, academic and research institutions, and nongovernmental organizations. Examples of initiatives in such a strategy include screening and surveillance of exposures, public education and awareness programs, environmental control of exposures, availability of adequate and accessible employee health services, worker safety programs, and medical programs aimed at protecting the health of all the citizens especially vulnerable populations namely children, elderly, and workers at risk. 

It is beyond the scope of this paper to discuss all of the above programs. Our discussion, which is aimed at primary care workers, presents relevant information that will encourage and allow primary health care workers to integrate essential components of occupational and environmental assessments in their day-to-day clinical and public health practice. While much of the information presented in this section applies to chemical exposures, we provide examples and illustrations related to metal exposures so as to stay focused on the theme of our paper. 

Why should frontline health care pay attention to occupational and environmental chemical exposures in the evaluation of their patients? Several reasons come to mind. One, exposures to hazardous chemicals is common [58–60]. In one primary care setting study, about 17% patients perceived their health problem being work related; 75% gave a history of prior exposure to one or more toxic agents [58]. In a recent study Pruss-Ustun and team estimate that of all deaths that occurred in 2004, 4.9 million were attributable to chemicals [3]. Two, most individuals who suffer from any problem including chemical exposure, make their first health contact with a primary care worker, who provides an initial evaluation of their problem. Many of these problems manifest as commonly occurring medical problems or nonspecific symptoms frequently seen in primary care situations. What is of concern is that many work-related problems including exposure problems are missed because primary care workers rarely consider and address occupational and environmental factors in their clinical evaluation [5, 61]. In general, clinicians' level of suspicion concerning environmental and occupational illness is usually very low [5, 58]. They do poorly when it comes to taking occupational exposure history [62]. Therefore, it is imperative that health care workers possess adequate knowledge and skills so that they can recognize early symptoms and signs of chemical toxicity, and when necessary refer them to experts and or agencies responsible for evaluating and managing chemical exposures. 

Any chemical exposure requires a comprehensive evaluation consisting of (a) obtaining a thorough medical and exposure history, (b) detailed physical examination, and (c) performing medical tests as might be indicated. Opinion of professional experts is invariably sought in documented exposures. It is not our intent to discuss the details of this type of evaluation. Instead, we focus on basic elements of exposure history that could be easily integrated in day-to-day routine primary care practice situations. Additionally, practical information on examination and tests that primary care workers could use in their day-to-day practice is provided. Any assessment tools and clinical protocols that are developed by practitioners should be based on the specific needs of those exposed, community environment, potential exposures, available resources and the level of training of health care workers. Information presented is general and can be incorporated in any clinical protocol used by primary care workers around the world.

### 6.1. Taking Exposure History: Basic Elements

Taking basic exposure history on every patient is important. Obtaining such history does not require detailed knowledge of toxicology. In seeking history the health worker should consider all possible exposures that may occur in the community where the patient lives and/or works. Exposure history can be done by asking a few questions or requiring patients to complete a simple form, the language of which should be simple and easily understood by the patient. Patient should be informed why exposure history is important. 

There are many occupational and environmental exposure history-taking approaches [5, 58, 62], available to clinicians. Most focus on obtaining the following information:

- current job of the patient—job title, type or nature of work, and any protective equipment on the job,
- patients perception whether or not their presenting symptoms are related to their work or the environment they live in,
- information on whether others at home or work present with similar problems,
- employment history and chronology of jobs held; temporal relationship is explored,
- relationship between work and health problems,
- environmental (nonwork) exposures—hobbies, smoking, household, herbal products, and community,
- specific environmental and/or occupational exposures—fumes, dust, metals, and chemicals,
- history of any comorbid conditions.

While all of the above information is vital, obtaining detailed time-consuming occupational and environmental history could be counterproductive [63, 64]. This especially applies to primary care situations where practitioners focus more on providing care to the presenting problem of the patient. 

Therefore, any environmental and occupational history taking approach should be designed such that it is easy to use and provides a snap shot of any exposures. One such approach that has been developed by the South Carolina Family Practice Residency programs uses the simple mnemonic WHACS [65]. It appears (verbatim) below:

W: what do you do?
H: how do you do?
A: are you concerned about any of your exposures on and off work?
C: coworkers or others exposed?
S: satisfied with your job?

Another initial and quick approach [58] focuses on the following four items:

1. kind of work patient does?
2. any relationship between work and health problems of the patient?
3. symptoms or problem better or worse at work or at home ?
4. any exposure to metals, chemicals, dusts, or fumes at home, work, or out in the community?

Additional questions can also be included to seek information on hobbies, use of herbal products, and exposure of coworkers or others at home.

Goldstein, in a recent Journal of Occupational and Environmental editorial, suggests even a simpler approach for occupational exposures [64]. The author recommends an initial question “What is your job?” followed by the “second question” which is “What is the riskiest part of your job?”. He argues that physicians have limited time and, in order to engage primary care workers in the initial evaluation of occupational health problems of their patients, the “second question” informs the patient that occupational health is important. The patient may respond identifying a particular risk which may then prompt the physician to ask a third question, “What are you doing to avoid that risk?”. The author suggests that this modest, empathetic, and interactive approach could be helpful in time-constrained primary practice situations. 

 While physical hazards such as radiation and noise are not the focus of this paper, questions on exposure to them could be incorporated in these approaches. Additional and detailed questioning may be warranted in some situations. The details of such questions are found in standard textbooks and references [5, 58] and other resources (Agency for Toxic Substances and Diseases Registry, Occupational and Safety Health Administration, and National Institute for Occupational Safety and Health websites). 

Since most occupational and environmental health care is rendered by primary care workers it is imperative that health care workers use simple and quick approaches to obtain exposure history. Examples of three such approaches are described above. Keeping the above guidance and principles in mind it should be easy to develop custom-tailored and novel approaches to meet the needs of the community and/or primary practitioners.

### 6.2. Examination and Medical Tests

Most primary care practitioners do not provide complete occupational and environmental exposure assessments and therefore do not require special skills to diagnose occupational and environmental health problems. However, some practitioners may benefit from practical information on examination and tests that they could use in their day-to-day practice in certain situations. 

Since most metals affect multiple organs and systems, it is recommended to conduct a complete systemic examination with a special focus on blood, cardiac, gastrointestinal, lung, liver, central nervous system, and kidney. Complete blood count, urine analysis, kidney function, and liver function tests are usually helpful. Chest X-ray and pulmonary function may be performed where relevant. Metallic content in blood, urine, and tissues may be used to confirm the diagnosis. Each metal produces a constellation of symptoms and a clinical picture unique to the metal. The tests required for exposures are metal specific. See Table 2 for routes of exposure, potential health effects, and specific tests required to diagnose and/or monitor the exposure. The information provided in Table 2 is not comprehensive, but it provides some general guidance on clinical aspects of exposure to arsenic, mercury, and lead—metals to which public and workers can be exposed. Medical and exposure history guides the nature of examination and tests to be performed.

### 6.3. Guidance and Referral

If there is a suspicion of metal related exposure or illness, it is vital to evaluate and manage the patient while taking necessary steps to prevent future exposures. It is beyond the scope of this paper to describe the details of relevant preventive and management strategies. However, it is worthwhile mentioning briefly that primary care workers could consider taking certain steps examples which include (a) counseling the patient aimed at prevention and health promotion, and treatment, (b) referring the patient to a specialist or designated health care facility, (c) partnering or collaborating with health care providers and/or government agencies, and (d) notifying their supervisors, appropriate government/environmental agency and/or employer as may be indicated. Various actions taken by primary care workers will depend upon their scope of practice, training and responsibility, availability of resources, nature of the problem/exposures, local laws, and patient needs and preferences.

## 7. Challenges

Exposures to metals cause significant mortality and illness. Failure to recognize occupational and environmental health problems in health care settings remains a challenge. Inadequate training of primary care practitioners and health care students in the occupational and environmental health disciplines is of concern. The priority given to these subjects in the medical and nursing schools curricula is very low. This must receive immediate attention. 

Long- and short-term exposures resulting in delayed onset of occupational and environmental illnesses will continue to defy scientists in better understanding the causes of such illnesses. Reports of presence of metals in various herbal products and their impact on human health are worrisome. The interaction between metal exposures and disease risk factors such as smoking and obesity will require a closer examination. Lack of adequate regulatory controls in many nations is also of concern. 

Establishing national registries for occupational and environmental health problems and investing in data collection and monitoring will require additional resources and intersectoral collaboration.

## 8. Conclusion

Exposure to chemicals is a serious public health problem that affects wildlife, soils, water, and air and can have very harmful human health effects. Exposures to chemicals including metals must be identified promptly, and individuals exposed to them must be evaluated and managed without delay. Vulnerable populations, namely, children, pregnant women, workers, and those at risk in community situations deserve our highest priority. Programs aimed at (a) providing basic occupational and environmental health education and training to health care workers, (b) creating public awareness about exposures to chemicals and their adverse health effects should be developed and implemented without delay. Ongoing epidemiological, public health, and clinical research on the subject will enhance our efforts in controlling metal exposures and contamination of the environment. Health care providers, scientists, academicians, environmental health departments, and employers must work together and make every effort to prevent human exposure to chemicals and metals. The problems associated with metals are not disappearing even as new control measures are implemented and more regulations are enforced. However, the lessons learned from the past may help limit the inevitable impact of heavy metal use as part of advancing industrialization in both less and more developed nations around the world.

## Figures and Tables

**Table 1:** Sources of exposure to arsenic, lead and mercury.

General population	Occupational populations
Arsenic	
(1) Air, drinking water, and food. Food is the predominant source. (2) Sawing and sanding, or burning of wood treated with arsenic-containing preservatives. Sawdust can be inhaled. (3) Arsenic also used in herbicides and as additives in animal feed.	(1) Workers involved in copper and lead smelting and wood treatment. (2) Workers who deal with arsenic-containing pesticides.

Lead	
(1) Lead-contaminated food and water, and also dust and lead paint. (2) Through foods from improperly glazed pottery. (3) Herbal remedies may contain lead. (4) Hobbies that use lead: soldering, making stained glass, and firing ranges.	Workers engaged in industries: lead smelting, battery manufacture, steel welding, construction, printing, radiator repair shops, rubber production, firing ranges, and printing.

Mercury	
(1) Dental amalgam fillings. (2) Practicing rituals that use mercury. (3) Damaged mercury-containing household items: thermometers, blood pressure devices, and fluorescent light bulbs. (4) Eating fish high in methyl-mercury. (5) Breathing contaminated air from hazardous waste sites that contain mercury. (6) Fungicides that contain mercury.	(1) Occupations in which there is a potential risk for mercury exposure—manufacture of electrical equipment, automotive parts that contain mercury, metal processing, and building parts and equipment that contain mercury (electrical switches, blood pressure devices). (2) Dentists and their assistants from breathing mercury vapor.

Adapted from References: [52–57].

**Table 2:** Routes of exposure, health effects, and diagnosis/medical monitoring for arsenic, lead, and mercury.

	Route of exposure	Health effects	Diagnosis/medical monitoring
Arsenic (inorganic and organic)	Inhalation, oral, dermal	(1) Acute exposure: nausea, diarrhea, GI bleeding, cardiovascular effects, shock, and death. Liver, kidney damage and seizures have been reported. (2) Chronic exposure: hyperpigmentation of skin, warts, corns, heart disease, neuropathy, liver damage, anemia and peripheral vascular disease (gangrene of lower limbs), and increased risk of skin, liver, lung and bladder cancer. Arsenic in drinking water can also cause diabetes and hypertension.	Urinary arsenic level is the most reliable indicator of recent exposure to arsenic. Arsenic in hair and fingernails can indicate exposure to high levels in the past 6–12 months.

Lead	Inhalation oral dermal	(1) Hematologic: decreased heme synthesis enzymes, anemia. (2) Cardiovascular: elevated blood pressure. (3) Cognitive, neurobehavioral, and psychological effects. (4) Gastrointestinal: colic or abdominal cramps. (5) Peripheral neuropathy; encephalopathy (at high levels). (6) Reduced fertility. (7) Immune system: alterations in T cell, reduced IgG serum levels. (8) Children: lethargy, loss of appetite, anemia, colic, neurological impairment, and impaired metabolism of Vit D. Exposure in uterus and during childhood can result in impaired neurological development, IQ deficits, and growth retardation. Lead-based paint is a common source of lead exposure.	Lead in whole blood is a reliable test. Erythrocyte protoporphyrin (EP) test can also be used but it is not sensitive to detect high levels of lead in children.

Mercury (elemental or metallic, organic-methyl mercury and inorganic)	Inhalation oral food (fish), dental work.	(1) All forms of mercury are toxic to the CNS. (2) Exposure to high levels can damage brain, kidneys, and developing fetus. (methyl mercury is the most toxic form). (3) Toxicity to brain results in irritability, tremors, visual changes, and memory problems. (4) Mercury salts can cause abdominal cramps, diarrhea, and kidney damage.	Acute exposure is best measured by mercury in blood and chronic exposure by mercury in urine.

Adapted from References: [52–57].
